# Exploratory biomarkers for acute rejection in vascularized composite allotransplantation

**DOI:** 10.3389/frtra.2026.1788046

**Published:** 2026-04-01

**Authors:** Dominika Pullmann, William J. Rifkin, Haruyuki Hirayama, Bruce E. Gelb, Ata S. Moshiri, Massimo Mangiola, Eduardo D. Rodriguez, Catherine P. Lu, Piul S. Rabbani

**Affiliations:** 1 Hansjörg Wyss Department of Plastic Surgery, New York University School of Medicine, New York, NY, United States; 2 Transplant Institute, New York University School of Medicine, New York, NY, United States; 3 Ronald O. Perelman Department of Dermatology, New York University School of Medicine, New York, NY, United States; 4 Department of Cell Biology and Regenerative Medicine Institute, New York University School of Medicine, New York, NY, United States

**Keywords:** biomarkers, reconstructive transplant surgery, reconstructive transplantation, rejection biomarkers, rejection monitoring, vascularized composite allotransplantation (VCA)

## Abstract

Vascularized composite allotransplantation (VCA) involves immunologically heterogeneous tissues with a high incidence of acute rejection. Reliable and timely detection of rejection onset remains a major unmet challenge in VCA management. This longitudinal exploratory case study assessed blood- and tissue-derived biomarkers for acute rejection monitoring in a full-face and bilateral hand transplant recipient over 4.6 years. Of these biomarkers, donor-derived cell-free DNA (dd-cfDNA) and short tandem repeats (STR) showed trends toward elevated recipient levels during acute rejection, though differences were not statistically significant. CD8^+^ T-cell percentages increased before acute rejection onset, highlighting a temporal association. Anti-angiotensin II type 1 receptor antibody (AT1R-Ab) levels did not differ significantly between acute rejection and non-rejection episodes, possibly due to prophylactic immune cell depletion. While dd-cfDNA and STR levels correlate with rejection episodes and reflect key graft cellular events, CD8^+^ T-cell dynamics demonstrated the strongest temporal association with rejection episodes in this patient, though no biomarker showed statistically significant differences. These exploratory findings support the need for further longitudinal, multi-patient studies to validate emerging biomarkers and refine rejection monitoring strategies in VCA.

## Introduction

Vascularized Composite Allotransplantation (VCA) involves transplantation of heterogeneous organs including skin, muscle, bone, nerves and vasculature. Skin is the most immunogenic component contributing to a rejection rate seven times higher than solid organ transplants (SOT) (1). Despite immunosuppression, up to 85% of VCA recipients experience acute rejection (AR), potentially progressing to chronic rejection and graft failure (2). Histopathological skin evaluation using Banff criteria, adapted from SOT, is the current gold standard for assessing rejection, but is limited by sampling variability, interpretive subjectivity, local inflammation, and cosmetic injury (3). Non-invasive imaging modalities such as MRI and ultrasonography can detect gross rejection-related changes but lack resolution for early microvascular or subclinical pathology (4). Deeper tissue rejection may lack visible signs, leading to insidious graft deterioration. No validated biomarkers reliably reflect rejection severity or predict AR before clinical manifestation. Evaluating alternative biomarkers for more timely and sensitive AR monitoring is crucial for improving VCA management and patient outcomes.

Unlike SOT, rejection detection in VCA is complicated by the varying immunogenic profiles of its constituent organs, particularly skin. Clinically, skin erythema and edema may signal early AR driven in part by CD8^+^-cytotoxic T-cell activity leading to keratinocyte exocytosis; however, the relative contribution of donor vs. recipient T-cells is incompletely defined (1). These clinical and mechanistic uncertainties limit conventional surveillance approaches and have prompted interest in molecular and cellular biomarkers as adjuncts to rejection monitoring.

Short tandem repeat (STR) profiling, used in solid and hematologic transplantation analyzes polymorphic repeat sequences that serve as distinct DNA signatures between donor and recipient. This technique quantifies recipient-to-donor DNA ratios to estimate recipient cell infiltration within grafted tissues (5, 6). Similarly, donor-derived cell-free DNA (dd-cfDNA) measured in recipient blood provides a measure of graft cellular injury and death, with prior SOT studies demonstrating elevations that precede clinical or histologic confirmation of AR (7–9). Both SOT and VCA studies have identified non-HLA-associated circulating antibodies, such as anti-angiotensin II type 1 receptor antibodies (AT1R-Abs), as potential biomarkers of rejection (10–12). Additionally, VCA literature has investigated the clinical utility of other biomarkers such as matrix metalloproteinase-3, circulating basophils, and C-reactive protein (13, 14). Together, these approaches represent promising yet incompletely characterized tools for rejection monitoring in VCA.

Given the central role of graft biopsies particularly early in our practice, we performed longitudinal STR profiling to not only determine the cell origin (recipient vs. donor), but also the percent contribution from each in the biopsy. Our own institutional experience with lymphocyte subsets and plasma dd-cfDNA in kidney transplant patients informed our assessment of cfDNA as a tool for VCA. In parallel, we selected AT1R antibodies as a non-HLA marker of graft injury based on their established relevance in SOT and the high expression of AT1R in skin, a principal component of VCA. In this context, we explored the longitudinal association of T-cell populations, STR profiling, dd-cfDNA, and AT1R-Abs biomarkers with AR within a single, complex VCA recipient.

## Methods

### Patient

**Figure 1:**
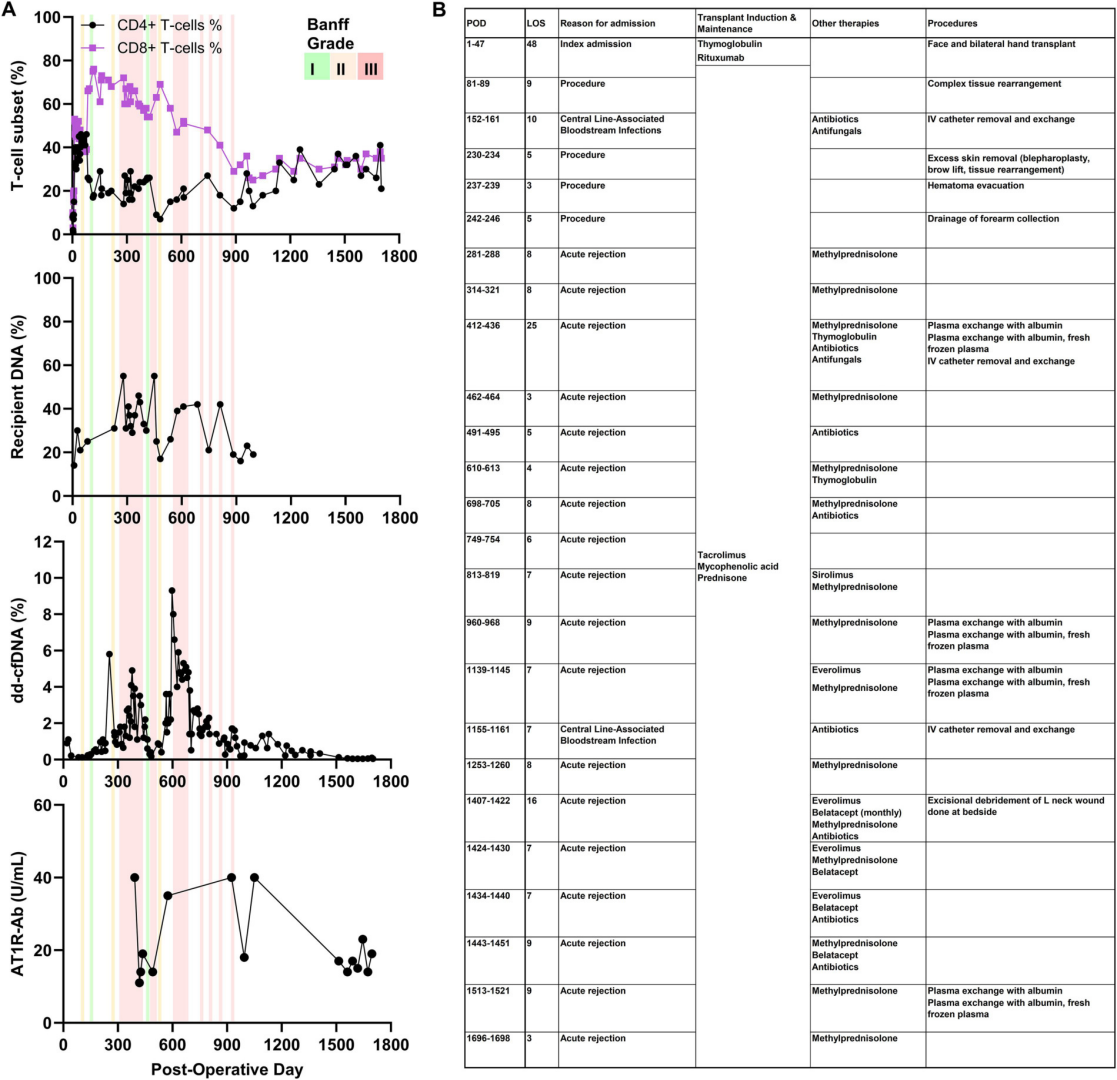
Post-operative rejection monitoring and management. **(A)** T-cell percentages (CD4^+^ and CD8^+^, each *n* = 83), percent recipient DNA in allograft tissue by STR profiling (*n* = 30), percent dd-cfDNA in plasma (*n* = 132), and AT1R-Ab levels (U/mL, *n* = 16). The absence of Banff data after POD 900 reflects the transition from routine biopsy-based diagnosis to clinical assessment. **(B)** Summary of treatments during inpatient admissions for revision procedures or AR. AR, acute rejection; AT1R-Ab, angiotensin II type 1 receptor antibody, dd-cfDNA, donor-derived cell-free DNA; LOS, length of stay; POD, post-operative day; STR, short tandem repeat.

We analyzed data from a male full face and bilateral hand allograft recipient in his 20s under NYU IRB approval (i19-00621). Clinical appearance, including edema and erythema, together with Banff grading of skin biopsies for immune infiltration and epidermal-dermal junction changes (15) defined rejection episodes. After approximately post-operative day (POD) 900, the diagnostic approach emphasized clinical assessment, incorporating examination and response to therapy with biopsies performed selectively to minimize cosmetic injury. Rejection treatment included intravenous steroids, plasmapheresis, and belatacept infusions (Figure 1B).

### Blood and allograft sampling

We evaluated samples across 4.6 years (up to POD 1700) (Figure 1A). We analyzed 83 samples for lymphocyte subsets by flow cytometry, 30 allograft skin biopsies for STR profiling, 132 plasma samples for percent dd-cfDNA content (Allosure, CareDx, CA), and 16 serum samples for AT1R-Abs (UCLA Immunogenetics Laboratory, CA) using ELISA (EIA-AT1RX, ThermoFisher, MA) reported as units/mL with positivity threshold >17 units/mL per manufacturer specifications. For STR profiling, we isolated DNA from tissue biopsies and performed multiplex amplification of polymorphic STR loci, followed by capillary electrophoresis and fragment analysis on genetic analyzers. We quantified non-shared alleles between donor and recipient at each locus and averaged them across loci to estimate their relative contributions.

### Statistical analysis

We considered samples collected outside of rejection-related inpatient hospitalizations as non-rejection (NR). Given repeated sampling from a single patient, all observations are non-independent and statistical comparisons are exploratory descriptions of temporal patterns within this individual rather than inferential tests. We used non-parametric Wilcoxon signed-rank tests comparing AR vs. non-rejection timepoints, with *p*-values reported descriptively. We performed all statistical analyses using GraphPad Prism 9.02.0.

## Results

### Lymphocyte subsets

**Figure 2:**
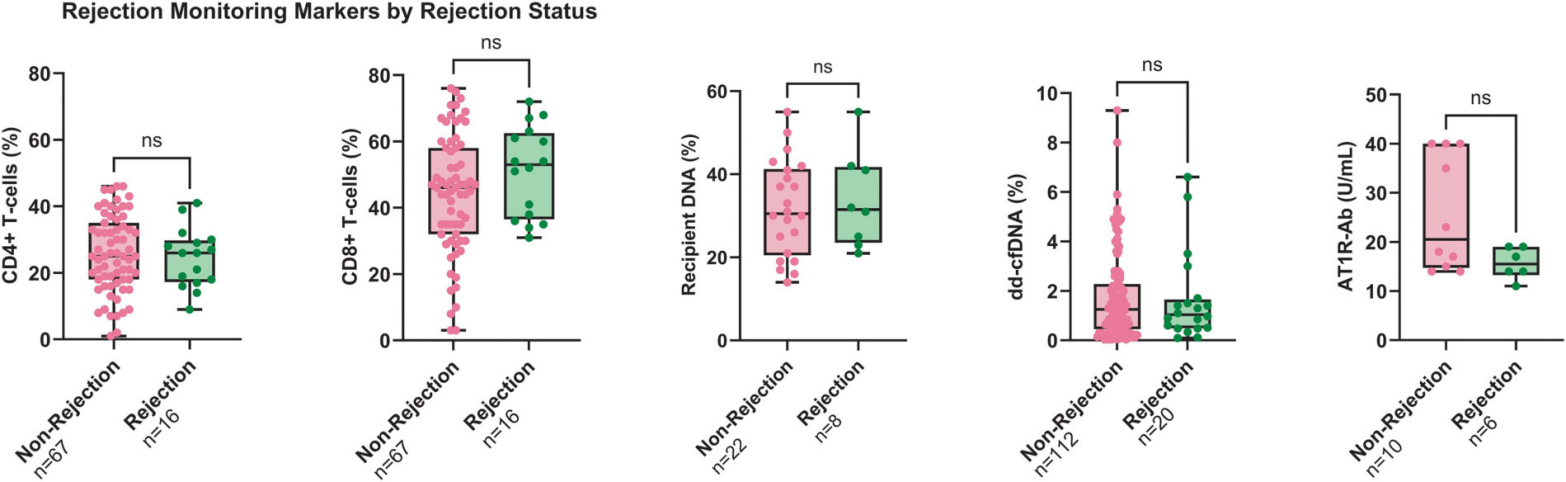
Rejection monitoring markers by rejection status. Box and whisker plots showing levels of five acute rejection markers during non-rejection vs. AR: CD4^+^ T-cell percentage (*n* = 67 vs. *n* = 16), CD8^+^ T-cell percentage (*n* = 67 vs. *n* = 16), percent recipient DNA in allograft tissue by STR profiling (*n* = 22 vs. *n* = 8), percent dd-cfDNA in plasma (*n* = 112 vs. *n* = 20), and AT1R-Ab levels (U/mL, *n* = 10 vs. *n* = 6). Boxes show interquartile range; lines denote medians; whiskers indicate full range. AR, acute rejection; AT1R-Ab, angiotensin II type 1 receptor antibody; dd-cfDNA, donor-derived cell-free DNA; STR, short tandem repeat.

The recipient CD3^+^ CD4^+^ T-cells represented a median of 25% overall of the post-transplant lymphocyte subset (IQR 18%–33%), 25% during non-rejection (IQR 18%–35%, *n* = 67), and 26% during rejection (IQR 17.25–29.75%, *n* = 16; *p* = 0.6741) (Figure 2). Recipient CD3^+^ CD8^+^ T-cells accounted for a median of 47% of lymphocytes post-transplant (IQR 35%–60%), 46% during non-rejection (IQR 32%–58%, *n* = 67), and 53% during rejection (IQR 36.5–62.5%, *n* = 16; *p* = 0.1509) (Figure 2).

Subgroup analysis of samples collected prior to beginning an mTOR inhibitor regimen (∼POD900) revealed similar recipient CD4^+^ T-cell percentages during non-rejection [25% (IQR 17%–38%, *n* = 51)] and AR [19% (IQR 16%–26%, *n* = 11; *p* = 0.1237)], but a trend towards higher CD8^+^ T-cell percentages during AR [60% (IQR 52%–67%, *n* = 11) vs. non-rejection 48% (IQR 44%–61%, *n* = 51; *p* = 0.0587)]. CD8^+^ T-cells increased more than CD4^+^ T-cells relative to their respective baselines, 164 days before clinical rejection (76% on POD117 from 39% on POD77), suggesting a temporal association preceding clinically defined rejection.

### Allograft skin STR profiling

STR analysis demonstrated a median recipient DNA content of 31% (IQR 22%–41%, *n* = 30) in graft biopsies, with no significant difference between non-rejection [30.5% (IQR 20.5–41.25%, *n* = 22)] and AR [31.5% (IQR 23.5–41.75%, *n* = 8; *p* = 0.6872)] (Figure 2).

### Plasma dd-cfDNA

Although dd-cfDNA appeared to correlate with AR episodes (Figure 1A), levels were similar between non-rejection [1.25% (IQR 0.45–2.275%, *n* = 112)] and AR [1.035% (IQR 0.48–1.65%, *n* = 20; *p* = 0.6949)] (Figure 2), with an overall median of 1.2% (IQR 0.48%–2.2%, *n* = 132). Despite an association, dd-cfDNA did not reliably distinguish between rejection and non-rejection.

### AT1R-Ab

Overall AT1R-Ab levels were 17.5 U/mL (IQR 14–32 U/mL), with no significant difference between non-rejection [20.5 U/mL (IQR 14.75–40 U/mL, *n* = 10)] and AR [15.5 U/mL (IQR 13.25–19 U/mL, *n* = 6; *p* = 0.1206)] (Figure 2).

## Discussion

This case study characterizes longitudinal patterns among exploratory surveillance biomarkers, including T-lymphocyte subsets, STR profiling, plasma dd-cfDNA levels, and AT1R-Abs, in a uniquely complex VCA recipient highlighting their potential roles and limitations in rejection monitoring. Given the rarity of VCA and the limited number of cases available for biomarker discovery and validation, we focused on markers measurable using clinical laboratory technologies previously established in SOT. This case describes our experience with these clinically available biomarkers in VCA rather than evaluating novel discovery-based markers.

### T-cell subset dynamics and early rejection

Consistent with the literature, T-lymphocytes increased during rejection periods (16). Notably, CD4^+^ and CD8^+^ T-cell subsets nearly doubled by POD117, almost six months before the first AR episode on POD281, detecting an earlier immune signal relative to clinically defined rejection in this patient. Subgroup analysis of samples collected before initiation of sirolimus suggested trending higher CD8^+^ T-cell percentages during AR, aligning with their role as cytotoxic mediators (16). Larger studies with more frequent sampling may clarify T-cell subset dynamics in VCA rejection.

### Interpretive limitations of allograft STR profiling

STR analysis showed a non-statistically significant increase in the percentage of recipient DNA within the allograft during AR, suggesting a potential, but unclear, role for infiltrating recipient immune cells in our patient (16). Biopsy-related graft injury and potential for sampling bias limit its clinical utility, as STR profiling can also reflect local immune infiltration rather than global graft status.

### dd-cfDNA trends and interpretive limitations in VCA

The association between dd-cfDNA and AR was not statistically significant, although observed trends are consistent with its proposed role as a rejection marker in SOTs (6–8, 17). Limited sensitivity and specificity in VCA may reflect unique skin-related factors including processing of keratinocyte DNA by tissue macrophages prior to systemic circulation and dd-cfDNA release from immunogenic skin during non-rejection stressors such as excess sun exposure, local trauma, or infection.

### AT1R-Ab levels in the context of B-cell depletion

AT1R-Ab levels did not differ between non-rejection and AR, in contrast to their established role in rejection in SOTs (10, 11). While preliminary, this suggests context-dependent utility as a biomarker, particularly with B/plasma cell depletion, as in our patient. While limited, this contributes to the emerging literature evaluating AT1R-Abs in VCA.

### Limitations and future directions

As a single-patient case study, these findings cannot establish diagnostic accuracy or clinical utility. Additionally, this dataset is limited by a small sample size for biomarkers and intermittent sampling that may have missed transient AR-associated biomarker fluctuations. Successful immunosuppression resulted in more NR sample-points than AR ones. Validating biomarkers requires larger cohorts, frequent sampling, and consideration of evolving VCA AR definitions. The evolution from histopathologic to clinical rejection criteria may introduce heterogeneity in outcome definitions, potentially affecting biomarker performance comparisons across different post-transplant periods. Importantly, none of the evaluated biomarkers demonstrated statistically significant differences by rejection status, and accordingly, their utility as reliable diagnostic or prognostic markers cannot be inferred from this limited dataset.

Despite these limitations, the evaluated biomarkers demonstrate the feasibility of longitudinal immune and injury surveillance in VCA. Lymphocyte subsets, STR profiling, plasma dd-cfDNA, and AT1R-Abs each capture distinct aspects of graft status, although their individual diagnostic utility for acute rejection remains limited. Integrating complementary biomarkers, potentially alongside advanced imaging or multi-omics approaches, may improve the sensitivity and interpretability of rejection monitoring in VCA.

In summary, although we found no statistically significant differences in biomarker levels between non-rejection and AR episodes within this limited dataset, this hypothesis-generating study provides longitudinal insight into rejection surveillance biomarkers in a complex VCA recipient. These exploratory findings help define the limitations and context of current biomarker strategies and underscore the need for larger, systematically sampled studies to test these preliminary observations and support biomarker development and validation in VCA.

## Data Availability

The datasets presented in this article are not readily available because the data contain information that could compromise the privacy of the research participant. Requests to access the datasets should be directed to Piul Rabbani at piul.rabbani@nyulangone.org.
